# Navigating Surgical Decision Making in Disorders of Sex Development (DSD)

**DOI:** 10.3389/fped.2018.00339

**Published:** 2018-11-19

**Authors:** Melissa Gardner, David E. Sandberg

**Affiliations:** Division of Pediatric Psychology and the Child Health Evaluation and Research (CHEAR) Center, Department of Pediatrics and Communicable Diseases, University of Michigan, Ann Arbor, MI, United States

**Keywords:** disorders of sex development (DSD), intersex, surgery, pediatrics, shared decision making

## Abstract

Surgical management of disorders of sex development (DSD) is associated with contentious debate between and within stakeholder communities. While the intent of surgical management of the genitals and gonads is to benefit the patient physically and psychosocially, these goals have not always been achieved; reports of harm have surfaced. Harm experienced by some patients has resulted in the emergence of an activist platform calling for a moratorium on all surgical procedures during childhood–excepting those forestalling threats to life within the childhood years. This ban is not universally endorsed by patient advocacy groups. Parents, meanwhile, continue to need to make decisions regarding surgical options for their young children. Constructive paths forward include implementation of Consensus Statement recommendations that call for comprehensive and integrated team care, incorporating mental health services, and adopting shared decision making.

## Background

The 2006 Consensus Statement on Management of Intersex Disorders introduced and defined “disorders of sex development” (DSD) as congenital conditions in which development of chromosomal, gonadal, or anatomic sex is atypical (1). DSD comprise many discrete diagnoses ranging from those associated with few phenotypic differences between affected and unaffected individuals to those where questions arise regarding gender of rearing, gonadal tumor risk, genital surgery, and fertility. Given their complexity, Consensus Statement recommendations for optimal DSD care include an experienced multidisciplinary team of pediatric subspecialists in endocrinology, surgery, and/or urology, psychology/psychiatry, gynecology, genetics, and neonatology; adding social work, nursing, and medical ethics, if available. Recommendations for a team approach were repeated in the 2016 update (2) which noted unresolved questions regarding genital and gonadal surgery performed before children are competent to provide informed consent. Evidence of controversy exists in the medical literature, social media, human rights organizations' deliberations, courts of law, and government legislatures. There is a drumbeat condemning, and calling for a moratorium on, elective genital and gonadal surgery without the individual's informed consent (3).

Given concerns raised over elective surgical interventions, the purpose of the present perspective is to: (1) revisit the use of terminology; (2) identify the rationale and expectations associated with early surgery; (3) examine current cross-cutting controversies and their implications for clinical management; and (4) suggest potential paths forward.

## Terminology

Understandings based on common terminology and classification are essential for scientific advancement (4, 5). DSD care is burdened by polarized terminology used to label or characterize conditions and procedures; e.g., surgery performed on the genitalia or gonads of infants or young children with DSD has been characterized by some as “infringements of bodily integrity and the rights of children” (6–9). “Cosmetic” and “medically unnecessary” are words used by opponents of early surgical interventions; “involuntary sterilization” for removal of the gonads. The choice of terms to describe DSD-related surgical procedures can provoke strong emotion, making it less likely for discussions to be balanced and rational.

Surgical interventions are generally classified as either *urgent* or *elective*. Urgent surgeries are performed promptly to avoid life-threatening circumstances or to prevent permanent disability. In DSD, urgent surgery may be needed to create unobstructed outlets for urine or stool. Elective surgeries include those that address non-urgent issues. A subset of these, cosmetic surgeries, are designed to enhance appearance without changing function. In DSD associated with atypical reproductive anatomy and/or genitalia, most surgical interventions fall in the elective category, but would not be considered exclusively cosmetic because altered function is the objective. A possible exception is early clitoral reduction surgery; the procedure would, in many cases, be categorized as cosmetic and carries risks to genital tissue sensitivity and adult sexual function and satisfaction (10). The Consensus Statement and clinical practice guidelines recommend surgery for clitoromegaly should only be considered in cases of severe virilization (1, 11, 12).

## Surgical interventions

DSD-related surgery involves procedures directed at the gonads, internal reproductive anatomy, or external genitalia.

### Gonadal surgery

Considerations of gonadal removal generally arise under two circumstances: reducing risk of gonadal tumors and avoiding contrasexual pubertal changes. Germ cell tumor risk is increased in DSD, but varies depending on the specific condition: risk is highest (30–50%) among patients with dysgenetic gonads containing Y chromosomal material and lower (<1–22%) for 46, XY conditions resulting from errors in testosterone biosynthesis or action (13–16). Contrasexual changes occur when the actions of pubertal hormones are at odds with the individual's gender identity. To prevent appearance changes from female to male (or male to female), the gonads may be removed before puberty if there is reasonable certainty that gender identity is firmly established and the person fully understands the implications of the procedure. If additional time is required to ascertain gender identity stability or competency in assenting, gonadotropin- releasing hormone agonist treatment may be prescribed to arrest pubertal development (17).

### Genital surgery

Genital surgery applies to procedures performed on external genitalia or internal reproductive structures. Beginning in the mid-1950s, the “optimal gender” principle guided clinical management of infants born with ambiguous genitalia. The principle considered multiple aspects of outcome, most prominently potential for complete sexual functioning (18). This approach was predicated on two assumptions (19): *gender identity* (i.e., self-identification as either girl/woman or boy/man) is not firmly established at birth, but rather is the outcome of gender of rearing; and stable gender identity and positive psychological adaptation require genital appearance match gender of rearing, which may involve reconstructive genital surgery. Both assumptions are contested (6, 20, 21).

Grounded in reports of surgical complications and dissatisfaction among some who had experienced early genitoplasty (22–25), intersex advocacy and human rights organizations condemn early DSD-related surgery unless medically urgent. A counterpoint is reflected in parents who recall strong wishes to surgically “normalize” their child's sexual anatomy in infancy and early childhood; they viewed genital surgery as “obvious” and “necessary” to assure their child's positive psychosocial and psychosexual adaptation (26, 27). This perspective is bolstered by follow-up studies of patients who had received early surgery, largely focused on women who had received early feminizing genitoplasty, suggesting predominantly favorable attitudes toward early feminizing procedures (28–31).

In a recent paper, Cools and colleagues stated “reconstructive surgery has always been a substantial part of DSD care and has remained so for many years seemingly without debate”; then noted “this has changed dramatically following disquieting reports of unfavorable outcomes, including high complication and/or reoperation rates and patient dissatisfaction” (32). In fact, it is unclear whether practices have changed (10, 33). The European Society of Pediatric Urology and Society for Pediatric Urology summarized their standpoint in a 2014 editorial as follows: “Atypically developed genitalia can affect not only physical appearance and body image, but also function of the urinary tract, kidneys, gonads, and the psychological and psychosexual development of the individual. Therapeutic management of these patients is, therefore, not limited to ‘cosmetic' surgery as stated in some reports…” Medical and surgical management aims were specified as: “Avoiding potential health hazards related to the altered anatomy and function of the urogenital tracts, meeting parents' expectations and helping the individual to achieve future satisfactory sexual function, consistent with their gender identity…” (34). In a review of outcome data focusing on surgical and sexual outcomes for patients with DSD, Lee and colleagues noted the goals of clinical management include a “surgical outcome with a good cosmetic appearance and functionality with potential for sexual intercourse with sufficient sensitivity for satisfactory responsiveness” (35). Lee also alluded to the importance of social factors interacting with surgery to influence patient quality of life, an issue emphasized more completely in studies conducted in non-Western societies (36–39).

## Controversy surrounding elective surgical intervention

Decisions about surgical procedures and their timing vary on a case-by-case basis and are contingent on the person's presentation and discussions with the family; yet the challenges to elective surgery on the gonads and genitals coming from activists are commonly categorical. The view that surgery on atypical genital and reproductive structures is necessary to deliver a desired appearance, capacity for sexual function, positive psychosexual development, and health- related quality of life is denounced by activists and some organizations. Evidence supporting (40–42) and ethics surrounding (43–47) surgical practices in DSD have been challenged. Critics of early elective surgery claim such interventions do not address the primary driving factors, i.e., parental anxiety, shame, and desire for secrecy regarding the child's sex anatomy (3, 48). Activists and providers recognize parents may feel stigmatized and seek to act quickly to “normalize” their child's appearance before becoming fully informed about all options and properly weighing risks and benefits of surgery (3, 49–51). Additionally, legal and ethical questions have been raised on the basis of patient autonomy (6, 44, 47, 52). Less often considered are the potential risks or comparative outcomes associated with performing surgery later in life (53, 54).

Recent years have seen a shift from calling for shared decision making (SDM) between parents and the young child's healthcare providers [e.g., (50)] to appeals for protecting the child's right to bodily autonomy and for the “right to an open future” (interpreted as a deferral of decisions regarding elective gonadal or genital surgery “until the patient himself/herself can participate meaningfully in decision making”) (55). Some activists who equate surgical intervention to “torture” worked with the UN High Commissioner for Human Rights and the UN Special Rapporteur on Torture and Other Cruel, Inhuman or Degrading Treatment or Punishment to call for the “prohibition of surgery and treatment on the sex characteristics of minors without informed consent” (7) and with state legislatures to limit genital surgery on children “until the child is able to participate in decision making”; e.g., California Senate Concurrent Resolution 110 (SCR-110) (56). There has also been a well-publicized legal case involving a young child with ovotesticular DSD in which plaintiffs claimed there had been inadequate informed consent for the surgery. The adoptive parents of the child were represented in court by the Southern Poverty Law Center (SPLC), an American legal advocacy organization specializing in civil rights and public interest litigation (57). The organization interACT—a nonprofit with “a focused mission of ending harmful medical interventions on intersex children” (58)—joined the SPLC in the lawsuit and supported SCR-110.

Patient support and advocacy organizations are not united in their positions toward early surgery. The US-based CARES (Congenital Adrenal Hyperplasia Research Education and Support) Foundation has been vocal in its condemnation of SCR-110. The most prevalent condition within the 46, XX DSD category is classic congenital adrenal hyperplasia (CAH); SCR-110 applies to this patient group. In an open letter at its website, the CARES executive director wrote “CAH is a life-threatening adrenal disorder, not a sexual disorder. CAH patients are not intersex. Therefore, SCR110 should not apply to CAH patients.” Rather than challenging the merits of the legislation, CARES Foundation dissociated itself from intersex and DSD.

An additional aspect of early elective surgery concerns potential harms of general anesthesia in early life. In December 2016, the U.S. Food and Drug Administration issued a “warning that repeated or lengthy use of general anesthetic and sedation drugs during surgeries or procedures in children younger than 3 years or in pregnant women during their third trimester may affect the development of children's brains” (59).

Although defended as therapeutic, early surgery in DSD is largely elective and, with limited exceptions, irreversible decisions could be postponed to an age when the minor is competent to be involved in discussions and provide assent without risking threats to physical health (47, 50, 60). Issues to consider are whether ultimate outcomes associated with surgery depend upon its timing. Performing genital surgery later (or not at all) may result in better, poorer, or comparable physical, psychosocial, and psychosexual outcomes. Presently, no framework for systematically collecting data in a prospective longitudinal manner on the effects of performing or withholding surgical interventions on infants and young children exists; nor does there exist a body of research that can speak to specific circumstances under which a minor would be considered able to make decisions about genital or gonadal surgery. Thus, no one has complete information on which to base decisions that carry life-long consequences for the child.

## Paths forward

Some activist organizations have urged governments worldwide to ban elective genital surgeries without the individual's informed consent (44). This suggests two future possibilities: one where all non-urgent procedures are eliminated until adulthood and one that leaves decisions to parents, providers, and patients—as they become increasingly able to provide assent as they mature (61).

### Surgical options become unavailable

The birth of a child with a DSD, and attendant uncertainty about the child's gender and psychological and sexual development, is considered extraordinarily stressful for parents (62). Many decisions made during this early period have permanent and far-reaching developmental consequences for the child. These challenges are compounded for families in which the DSD is a consequence of a chronic and life-threatening medical condition (e.g., classic CAH) (63). Families who desire early surgery, but live in jurisdictions where surgical management is curtailed, may experience increased distress, feelings of shame, and maintain intensified secrecy about their child's genital difference (3).

Availability of psychosocial interventions targeting parental efficacy in managing challenges for themselves or for their child will be critical—as will services for the children themselves. Indeed, advocates calling for a moratorium on surgery for those <18 years old have also called for a robust patient- and family-centered approach to care in which psychological services are essential (3). Given documented harm resulting from secrecy, psychosocial interventions designed to promote open and developmentally appropriate information sharing with the child must be implemented (64).

One difficulty in applying psychosocial interventions as an adjunct or alternative to surgery is that treatment specific to the needs of patients with DSD and their families have not yet been developed and demonstrated efficacious. This does not mean there is nothing to offer: cognitive- behavioral and problem-solving psychosocial interventions have demonstrable efficacy in improving psychosocial functioning of patients and families in other pediatric conditions which could be translated to DSD. A second difficulty derives from the limited availability of providers with specialized training to implement these interventions. Healthcare systems and centers have not fully implemented Consensus Statement recommendations to include behavioral health providers as full members of multidisciplinary teams (65). Healthcare systems are frequently found not to offer adequate funding for complex multidisciplinary care (66). System-level changes are needed to implement these changes (3).

Previous work of patient advocates has affected clinical management in positive ways: promulgating openness with patients and parents about all aspects of the child's condition, acceptance of shared decision making as an element of patient- and family-centered care, and increasing healthcare systems' accountability with regard to providing effective psychosocial services (3). It is hoped that continued coordination between providers and patient advocacy organizations will help in advancing changes recommended in the Consensus Statement with regard to behavioral health.

### Surgical options remain available

In 2018, the American Academy of Pediatrics (AAP) reaffirmed its endorsement of “patient- and family-centered care”—a term intended to explicitly capture the importance of engaging the family and patient as essential healthcare team members. Core principles include: listening to and respecting each child and family; ensuring flexibility in organizational policies, procedures, and provider practices so services can be tailored to the needs, beliefs, and cultural values of each child and family; sharing complete, honest, and unbiased information with patients and families on an ongoing basis so they may effectively participate in care and decision making to the level they choose; ensuring formal and informal support (e.g., peer-to-peer support) for the child and family; collaborating with patients and families at all levels of healthcare; and recognizing and building on the strengths of individual children and families and empowering them to discover their own strengths, build confidence, and participate in making healthcare choices and decisions (67).

Parents of young children with DSD are responsible for making decisions on behalf of their child. However, they often do not recognize that there are decisions to be made, nor always appreciate their role in a shared decision making (SDM) process; e.g., in the aforementioned study in which many parents characterized surgery as obvious and necessary, they did not experience it as something that involved decision making (26). These and similar observations (27, 68) strongly suggest a role for employing a SDM approach to educate and guide parents in working with clinicians. The objective of SDM is to help patients (or, during infancy and childhood, patient-surrogates; i.e., parents) make informed, preference-based clinical management choices among several relevant options (69).

SDM comprises three essential elements: explicit acknowledgment that a decision is required; the best available evidence concerning the risks and benefits of each option are reviewed and understood; and the process takes into account the patient's/family's values and preferences together with the provider's guidance (70, 71). SDM does not imply providers and patients/parents must have equal responsibility for the final decision (70, 72), nor that decisions are based entirely on patient preference; rather, it combines providers' expert knowledge and patients'/parents' rights to make healthcare decisions with full information; it requires involvement of providers and patients/parents, with bidirectional information exchange, mutual deliberation on treatment options, and agreement on treatment plans (73, 74).

In DSD, the lack of readily accessible information for parents poses a significant barrier to SDM. Parents have expressed their desire for a “survival guide or playbook” to explain their child's condition to them in understandable terms and practical information (75). Parents have reported not receiving adequate information regarding their child's condition and felt uncertain about the expected appearance of their child's genitals after surgery (76). Lack of clear information was noted as one of the most stressful and frustrating aspects of parenting a child with a DSD (26). Poorly informed decisions (e.g., those in which the complexities inherent in DSD-related clinical management decisions are not routinely and systematically presented to parents) or in which decisional conflict is present, represent risk factors for decisional regret (77). It is in particular under such circumstances—conditions of uncertainty—that SDM may be most beneficial (78, 79).

Decision-support tools (DSTs) reflect a strategy to promote a SDM process between healthcare providers and patients (or parent surrogates). Differing from traditional health education materials, DSTs are designed to help people make deliberate choices among options by explicitly describing treatment choices; providing quantitative risk and benefit estimates, when available; tailoring information to individual patients; and providing a context in which patients and parents can consider treatment options in light of their own values (80, 81). Through its process-driven stages, SDM has the added potential benefit of increasing the use of relevant evidence by providers in the course of usual care.

**Figure 1:**
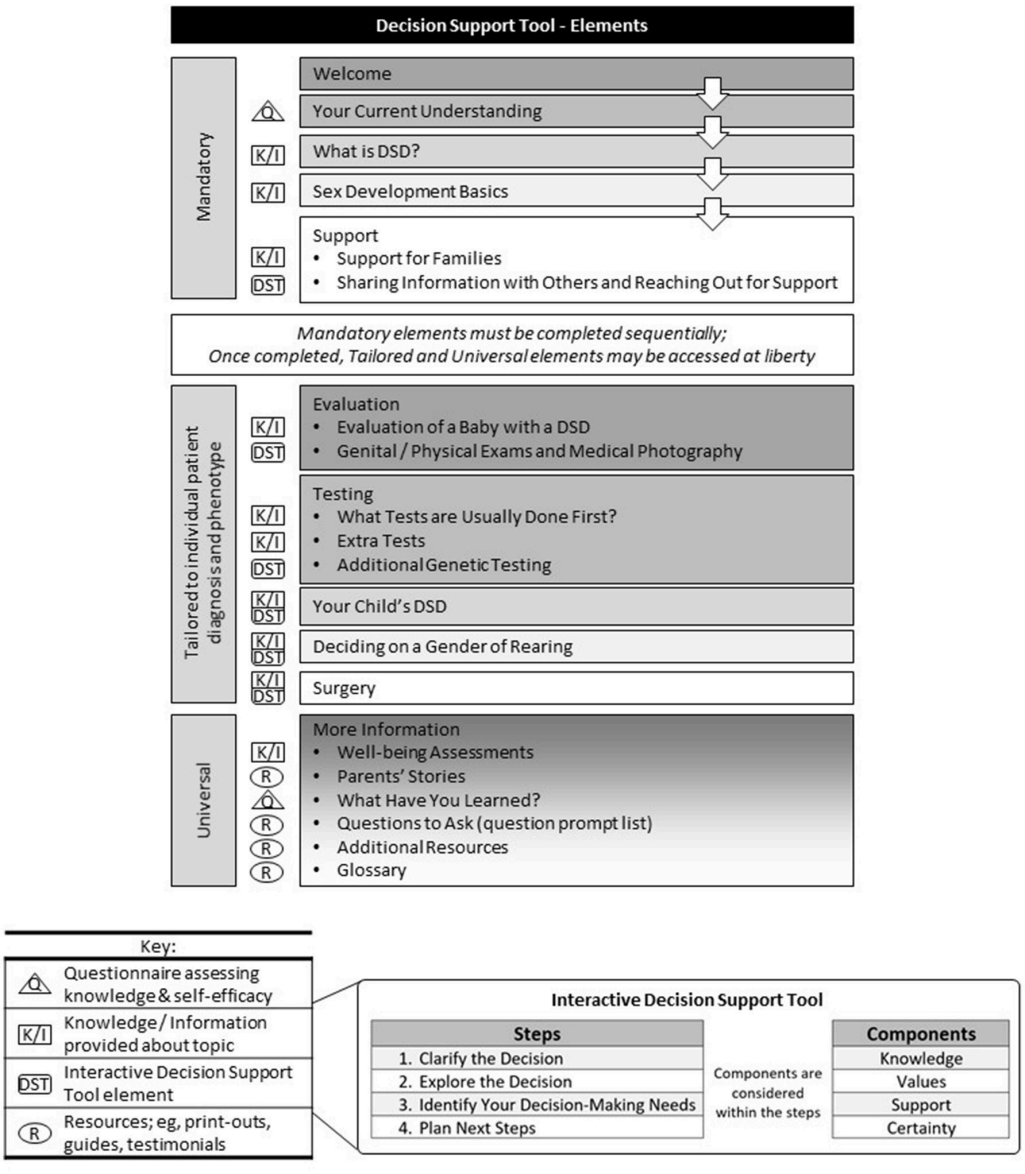
Decision Support Tool Elements.

Studies of SDM in adult patients have shown the vast majority want to be offered choices about their care and asked their preferences (82). Yet roughly half desire their provider make the final decision (82). Given the controversies surrounding early surgery in DSD, pediatric surgeons might understandably experience trepidation when the child's parents rely on them for the ultimate decision. The process of engaging patients (or their surrogates) more closely in clinical decisions may result in their increased willingness to declare their wishes. Although their choice may not align with the provider's viewpoint of the optimal action, the decision is more likely the one to which the family is prepared to commit. Work is currently underway on the development of DST for the parents of infants or young children with DSD and their providers (69) (see Figure 1). The DST was designed with input from multiple stakeholder groups, including providers who went on to pilot its use (69, 83). Results from the pilot project, which mirror other work in which DST were introduced to support SDM (84–86), suggest several factors must be in place to for it to succeed. These include motivation and “buy-in” from patients (and parents) and providers.

#### Patients (and parents)

As previously noted, parents are often unaware that any decisions around surgery need to be made and that they are key participants in that decision. In this vein, providers can use the DST not only as a means of providing information, but as a way of engaging patients/families in the SDM process. Additionally, just as it is important for physicians to maintain documentation of clinical management decisions, such a record is also important for patients to be able to reflect on their previous decisions or those of their parents and physicians had decisions occurred prior to their ability to be fully involved. For this to work, barriers such as overcoming technical problems when accessing web-based content and misunderstanding the importance of their role in SDM need to be addressed.

#### Providers

In an era when controversy has led to situations in which legislative bodies, rather than physicians and parents make proxy clinical care decisions on behalf of young patients (e.g., SCR-110), DST offer a standardized process by which patients (and parents) and providers participate in documented shared decision-making. However, the notion that “we already do shared decision-making,” (i.e., the belief that the providers are already sharing in the decision-making process and that a DST is superfluous) needs to be recognized and addressed. Additionally, organizational factors reducing providers' motivation to adopt SDM, including the perception that it will be time consuming or otherwise interfere with provider workflow need to be problem-solved (87). Meaningful integration of a DST into the workflow of comprehensive DSD care is not only predicated on the providers' expectations that the tool serves as a means of delivering patient- and family-centered care, but on the flexibility of organizational factors that can limit or enhance the ability of providers to use a DST. Finally, beyond the provider's commitment, training in SDM and the use of a DST is necessary (88, 89).

## Concluding comments

The role of surgical intervention in DSD is contentiously debated. It has been assumed that “normalizing” appearance and function and forestalling physical and psychosocial morbidity are goals, and known outcomes, of surgical intervention. Adults have reported satisfaction that their genital surgery had been performed early; conversely, reports of harm also exist. It is difficult to know how representative these experiences are. Gaps in high quality evidence that could be used to inform decision making on individual and healthcare policy levels is attributable to a number of factors: rarity of the conditions, heterogeneity of presentations, attrition of patients in follow- up from childhood to adulthood, and long intervals between surgery and time of data collection (32). Provided elective surgical intervention remains a part of DSD clinical management, registry-based research efforts, such as those of the European I-DSD/I-CAH (90) and US DSD- TRN (91), will provide important insights into the relationships between treatment options—surgical and non-surgical—and patient outcomes. In the interim, further integration of behavioral health services in DSD teams, buttressed by implementing robust SDM processes, is warranted. Development and effective application of DSTs in the clinical context is an area in which patient advocates can collaborate with healthcare providers.

## Author contributions

MG and DS identified relevant elements to include in this perspective. MG outlined and completed the first draft of the manuscript. DS revised the manuscript. Both authors collaborated on final edits.

### Conflict of interest statement

The authors declare that the research was conducted in the absence of any commercial or financial relationships that could be construed as a potential conflict of interest.
